# Differential placental ceramide levels during gestational diabetes mellitus (GDM)

**DOI:** 10.1186/s12958-019-0523-6

**Published:** 2019-10-23

**Authors:** Juan F. Mejia, Kelsey M. Hirschi, Kary Y. F. Tsai, Matthew G. Long, Benton C. Tullis, Eliza E. K. Bitter, Benjamin T. Bikman, Paul R. Reynolds, Juan A. Arroyo

**Affiliations:** 1 Department of Physiology and Developmental Biology, Lung and Placenta Research Laboratory, Brigham Young University, 3052 LSB, Provo, UT 84602 USA; 2 Department of Microbiology and Molecular Biology, Brigham Young University, Provo, UT USA; 3 Department of Physiology and Developmental Biology, Metabolism Research Laboratory, Brigham Young University, Provo, UT USA

**Keywords:** Trophoblast, GDM, Ceramide, Insulin

## Abstract

**Background:**

Gestational diabetes mellitus (GDM) is associated with important factors that influence fetal development. Sphingolipids are known to be associated with the development of diabetes. Our objective was to examine ceramide, a key sphingolipid, hyperosmolarity, and apoptosis in placentas from GDM patients treated with insulin or diet.

**Methods:**

Ceramide levels were assessed in placental tissues using immunohistochemistry. Immunoblot was performed to quantify serine palmitoyltransferase (SPT), the rate-limiting enzyme in ceramide biosynthesis, NFAT5, SMIT, AR, caspase 3 and the X-linked inhibitor of apoptosis. Trophoblast cells were treated with insulin or ceramide and assessments for mitochondrial respiration, caspase 3 and XIAP were also performed.

**Results:**

Immunohistochemistry showed increased ceramides in the placental villous trophoblasts of the insulin-treated GDM patients. Nuclear SPT was upregulated only in the insulin-treated GDM placenta when compared to controls. Nuclear NFAT5 was also increased in the GDM placenta. Active caspase 3 was elevated in placentas from both insulin- and diet-treated GDM patients. Mitochondrial respiration was decreased in trophoblasts treated with ceramide. Active caspase was not changed while XIAP protein was increased in trophoblasts treated with ceramide.

**Conclusions:**

Our findings confirm the presence of ceramide in the human placenta of control and GDM patients. Furthermore, we conclude that ceramide is increased in the placental trophoblast during insulin treatment and that its upregulation correlates with elevated NFAT5, SMIT, increased apoptosis and decreased trophoblast mitochondrial respiration.

## Background

Maternal-fetal interactions mediated by the placenta are critical for fetal development and overall positive outcomes during pregnancy. During gestation, the placenta is responsible for mediating the interface between mother and fetus where it functions to regulate processes such as gas exchange, nutrition availability, and waste removal [1]. Within the placenta, trophoblasts are an essential cell population that confers benefits during the development of the fetus as normal trophoblast function is necessary in the formation of a functioning placenta. One particularly pertinent feature of the trophoblast is its involvement in nutrient transport, an important step for proper fetal development [2]. Importantly, aberrant trophoblast function has been implicated in several pregnancy complications, including gestational diabetes mellitus (GDM) [1, 3–5].

While pregnancy elicits an inherent, progressive insulin-resistant state, GDM is a state where the pregnant body has become sufficiently resistant to the glucose lowering effects of insulin that hyperglycemia develops [3]. This pathology affects up to 12% of all pregnancies and can lead to higher risk of short- and long-term maternal and fetal complications. Importantly, GDM is expected to rise in the coming years [6, 7]. The list of maternal and fetal complications associated with GDM is lengthy, including maternal gestational hypertension and preeclampsia, shoulder dystocia, caesarian delivery, hyperglycemia in the infant, and the development of type 2 diabetes for both mother and child [8]. The GDM placenta is characterized as one of increased size, inflammation, and angiogenesis, while decreased trophoblast apoptosis and sporadic cases of increased placental osmolarity has also been indicated [3, 7]. Recently, insulin resistance was also shown to be associated with decreased placental efficiency [9]. Often diet, exercise or insulin are used to reduced GDM complications and to induce proper glycemic control in pregnant mothers [6].

Recent work showed differential lipid levels in the serum of mothers affected by GDM [6]. In particular, research showed a varying sphingolipid profile in the serum from mothers affected by GDM when compared to control, non-GDM mothers [6]. Besides being part of the plasma membrane, sphingolipids also regulate cellular activities such as cell survival, differentiation and proliferation [10]. Ceramide is a primary sphingolipid, considered the “backbone” of downstream sphingolipids, widely studied for its role as an effector molecule in the cellular response to stress and apoptosis, which can also be affected by hyperosmolar stress [10–12]. Ceramides are primarily generated by de novo synthesis in the endoplasmic reticulum through the enzymatic effects of the serine palmitoyltransferase enzyme (SPT) [11, 13]. Ceramide is expressed in the placenta and a plausible role for ceramide in the placenta was recently described in relation to controlling trophoblast syncytialization [10, 11]. Thus, ceramides may be a meaningful mediator in GDM-related placental pathologies; not only does ceramide disrupt nutrient transport, including amino acids and glucose [14], but ceramides also induce insulin resistance, increasing the risk of non-insulin dependent diabetes mellitus [15].

We have more recently found that ceramide accrual forces deleterious mitochondrial changes that may be relevant in altering placental physiology [16]. Despite the apparent associations between GDM and disrupted trophoblast function as a source of GDM-related placental pathologies, the relationship between these variables, including the potential role of ceramides, remains vague. Thus, the purpose of this study was twofold. First, our objective was to investigate the osmolarity factors in gestational diabetes, where we see significant glucose shifts that may contribute to large variations in osmolarity and placental ceramide accumulation in control and treated conditions in humans. Diabetic treatment involving insulin (GDM-I) vs. dietary interventions (GDM-D) both alter ceramides [17]. Second, we wanted to establish the effect of ceramides on trophoblast mitochondrial bioenergetics and cell invasion. Together, these studies help elucidate clear association between GDM, placental ceramides, and trophoblast function.

## Materials and methods

### Placental biopsies and paraffin embedded tissues

Placental biopsies and slides from paraffin embedded placental tissues for GDM-I (gestational diabetes mellitus treated with insulin), GDM-D (gestational diabetes mellitus treated with diet), and term controls (non GDM healthy pregnancy) were obtained from the Research Center for Women’s and Infant’s Health Biobank, Ontario, Canada. These samples were collected immediately following normal vaginal or cesarean deliveries from uncomplicated term gestations (*n* = 5).

### Immunohistochemistry

Immunohistochemistry (IHC) was performed for ceramide localization in the placenta as previously performed in our laboratory [18]. Briefly, placental slides (*n* = 6) were deparaffinized, washed in TBS and blocked for 30 min with Background Sniper (Biocare Medical, Concord, Ca). Slides were incubated for 1 h with a mouse monoclonal primary antibody against cytokeratin 7 (for trophoblast localization; Dako, Carpinteria, CA), ceramide (R&D Systems, Minneapolis, MN) or with a universal IgG negative control (Biocare Medical; Concord, CA). Sections were incubated with Mach 2 secondary antibody (Biocare Medical, Concord, CA). Slides were developed with diaminobenzidine (DAB) for cytokeratin 7 or ceramide. Slides were imaged at 20X magnification.

### Immunohistochemistry quantification

Individual images were analyzed using imageJ software when evaluating the staining intensity of external peripheral tissue for controls (ceramide and isotype) and treatments (GDM-D and GDM-I) [19]. ImageJ images were quantified by first filtering for DAB specific staining and then subsequently images were converted to a grayscale for analysis [20]. A universal threshold was applied to the tissue to eliminate areas of negative space from the analysis. The membrane of each treatment (GDM-D and GDM-I) was measured (*n* = 10) and subsequently quantified by assessing the mean gray value across each membrane; of note, the lower the gray intensity the darker the staining.

### Cytoplasmic and nuclear extraction

Nuclear and cytosolic proteins were extracted from placental biopsies from GDM-I, GDM-D, and control samples using the NE-PER nuclear protein extraction kit (Pierce, Rockford, IL). Briefly, 100 mg of placental tissues was weighed, placed in 500 μl of cytoplasmic extraction reagent I (CER I) and homogenized; 27.5 μl of CER II were added to the samples, vortexed, and incubated on ice for 1 min. Samples were spun and the pellets were resuspended in 125 μl of ice-cold nuclear extraction reagent (NER). The samples were vortexed and returned to ice and vortexing continued for 15 s every 10 min for a total duration of 40 min. Samples were centrifuged, and the supernatant (nuclear protein) was transferred immediately to a pre-chilled tube and placed on ice. When not used immediately, all extracts were stored at − 80 °C. The quality of the extraction was tested by Western blotting of both the cytoplasmic and the nuclear extracts with antibodies against lamin B (a nuclear housekeeping protein, Santa Cruz Biotechnology, Dallas, TX) or actin (Abcam, Cambridge, MA).

### Western blotting

Control, GDM-D, and GDM-I samples were obtained from the Research Centre for Women’s and Infant’s Health Biobank. Immunoblotting was performed as previously done in our laboratory [21]. Whole tissue lysates (50 mg) or cytoplasmic and nuclear extracts lysates were loaded (15 mg of protein) and separated on 4–12% Bis-Tris Midi Gel (Novex by Life Technologies, Carlsbad, CA). Proteins were transferred to nitrocellulose membranes using Invitrogen iBlot (Novex by Life Technologies, Carlsbad, CA). For protein determination, membranes were blocked in 5% milk in TBST for 1 h followed by overnight incubation with primary antibodies against: mouse NFAT5 (Affinity Bioreagents, Golden, CO), mouse SLC5A3 (SMIT; Fisher Scientific, St. Louis, MO), rabbit AR (Santa Cruz Biotechnology, Santa Cruz, CA) serine palmitoyltransferase 1 (SPT1, Sigma-Aldrich, St. Louis, MO), active caspase 3 (Cell Signaling, Danvers, MA), XIAP protein (an inhibitor of caspase activation Abcam, Cambridge, MA) Lamin B1 (Santa Cruz Biotechnology, Dallas, TX) or beta-actin (Abcam, Cambridge). Membranes were incubated with a secondary anti-rabbit horseradish peroxidase (HRP) conjugated antibody (Pierce Biotechnology, Rockford, IL,) for 1 h at room temperature followed by development using ECL substrate (Advansta, Menlo Park, CA). Proteins were detected by exposure of membranes to X-ray film and development. The presence of these proteins was confirmed and quantified. Bands were analyzed digitally with AlphaEaseFC software (Alpha Innotech Corporation, San Leandro, CA).

### Cell culture and treatments

Human BeWo choriocarcinoma cells (which have a villi syncytiotrophoblastic phenotype) were maintained in F12K media supplemented with 10% fetal bovine serum (FBS) and 1% penicillin and streptomycin. Cells were plated at a density of two hundred thousand cells per well cm in six-well plates. Cells were treated with C2-ceramide (1 μM; Sigma-Aldrich, St. Louis, MO), insulin (50 nM, Sigma-Aldrich, St. Louis, MO) or fresh media for 24 h. Importantly, C2-ceramide is an oft-used agent, due to its solubility. After treatment, BeWo cells were used for mitochondrial respiration determination. Cell lysates were collected and evaluated for active caspase 3 and XIAP immunoblot determination.

### Mitochondrial respiration

High-resolution O_2_ consumption was determined at 37 °C in permeabilized BeWo cells using the Oroboros Instruments O2K oxygraph. Before the addition of the samples into the respiration chambers, a baseline respiration rate was determined. After addition of the sample, the chambers were hyperoxygenated to ~ 350 nmol/ml. Following this step, electron flow through complex I was supported by GM (glutamate+malate; 10 and 2 mM respectively). Following stabilization, ADP (2.5 mM) was added to determine oxidative phosphorylation capacity (GMD). The integrity of the outer mitochondrial membrane was then tested by adding cytochrome c (10 μM; not shown). Succinate was added (GMSD) for complex I + II electron flow into the Q-junction. To determine the full ETS (electron-transport system) capacity over oxidative phosphorylation, the chemical uncoupler FCCP (carbonyl cyanide p-trifluoromethoxyphenylhydrazone) was added (GMSE; 0.05 μM).

### Statistical analysis

Data are shown as mean ± SE. Differences between groups were determined using Krauskal-Wallis test, with *P* < 0.05 considered significant.

## Results

### Maternal demographics

Demographics of human placental sample donors were analyzed for significant differences between control (non GDM normal health pregnancy), GDM-D and GDM-I groups. There were no significant differences in maternal age, BMI, gestational weeks and fetal weight between control and both GDM pregnancies (Table 1).

**Table 1:** Patients Demographical Data from collected placental samples

	Control	GDM-D	GDM-I	*P* value
Maternal Age	33 ± 2.1	35 ± 1.4	34 ± 1.6	0.772
Gestational Age (wks)	39 ± 0.07	38 ± 0.3	39 ± 0.8	0.776
Fetal Weight (g)	3498 ± 59	3245 ± 151	3453 ± 217	0.306
% C-section /Vaginal	90%/10%			

Groups (*n*=5) were analyzed for statistical significance (*p* < 0.05) using the Kruskal-Wallis test. There was no difference in maternal age, gestational age and fetal weight between control and GDM pregnancies

### Placental ceramide levels and SPT expression

**Fig. 1:**
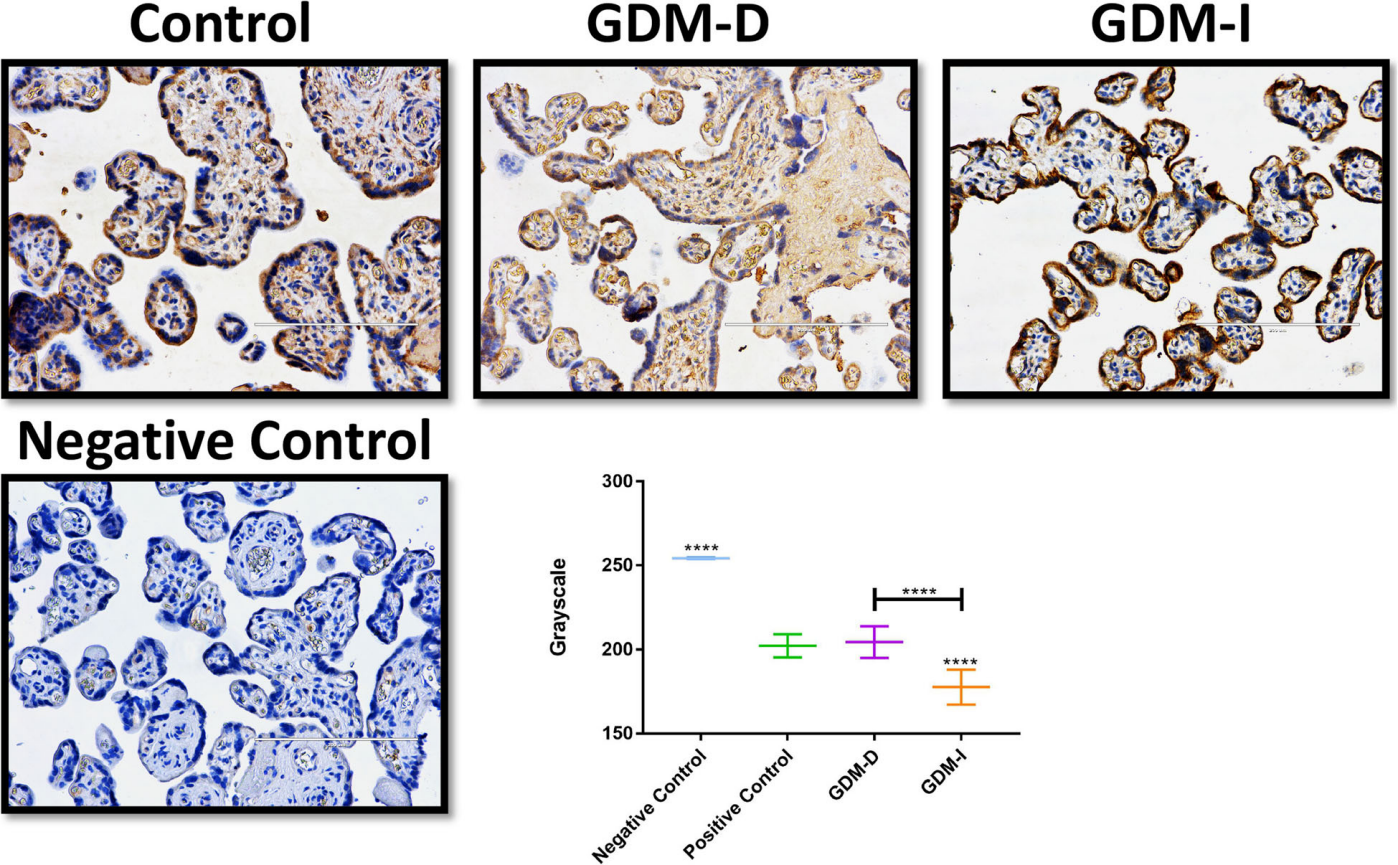
Ceramide and cytokeratin levels in the control and GDM placentas. Immunohistochemistry shows that ceramide is elevated in trophoblast cells that also express from GDM-I placentas and decreased in the GDM-D placentas compared to controls. Original images were imaged at 20X and scale bars are 50 mm. Imaging quantification confirm these results

Ceramide is present in the villi of trophoblast cells [10, 11] so we investigated ceramide levels in control placentas and GDM placentas induced with either diet or insulin. A set of representative images of ceramide staining is shown in Fig. 1. Immunohistochemistry quantification confirm increased ceramide staining in the villous trophoblast of the placenta during GDM-I but not in the GDM-D tissues (Fig. 1).

**Fig. 2:**
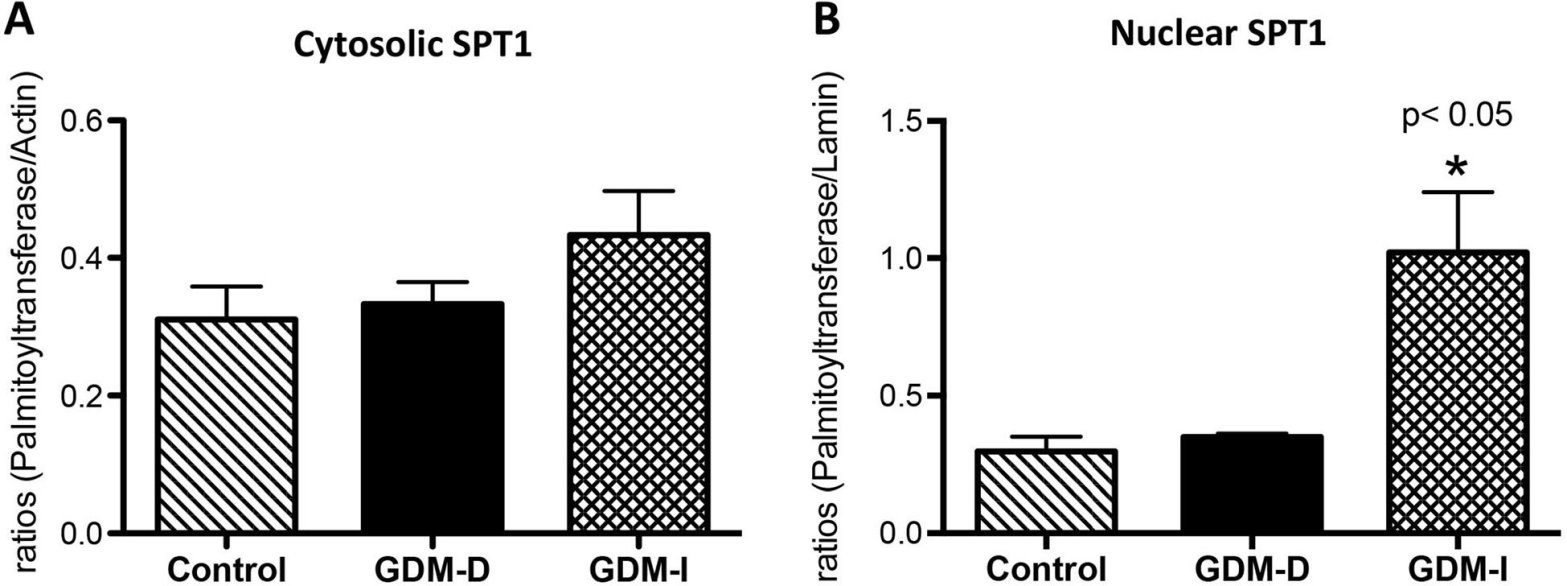
Serine palmitoytransferase 1 in control and GDM human placentas. Cytosolic and nuclear levels of serine palmitoytransferase 1 (*n* = 5) was measured by western blot and quantified by Spot Denso analysis; histograms show mean ± SEM. Cytosolic serine palmitoytransferase 1 levels were not changed in the GDM-D or the GDM-I placentas when compared to control samples (**a**). Nuclear serine palmitoytransferase 1 levels were elevated in in GDM-I (*p* < 0.05) placenta when compared to control placenta samples (**b**). Experiments were conducted in triplicate and statistically different values are noted as * *p* < 0.05

We next wanted to investigate the degree to which the de novo ceramide biosnynthetic pathway was affected. Thus, we explored SPT1 levels, one isoform of the rate-limiting biosynthetic enzyme [13]. No significant differences were observed for cytosolic SPT1 expression between control and GDM placental tissues (Fig. 2a). In contrast, highly upregulated expression of the nuclear SPT1 enzyme was present only in the GDM-I placenta (3.4-fold; *p* < 0.05) when compared to controls (Fig. 2b), highlighting the potential relevance of a nuclear source of ceramides.

### Hyperosmolarity

**Fig. 3:**
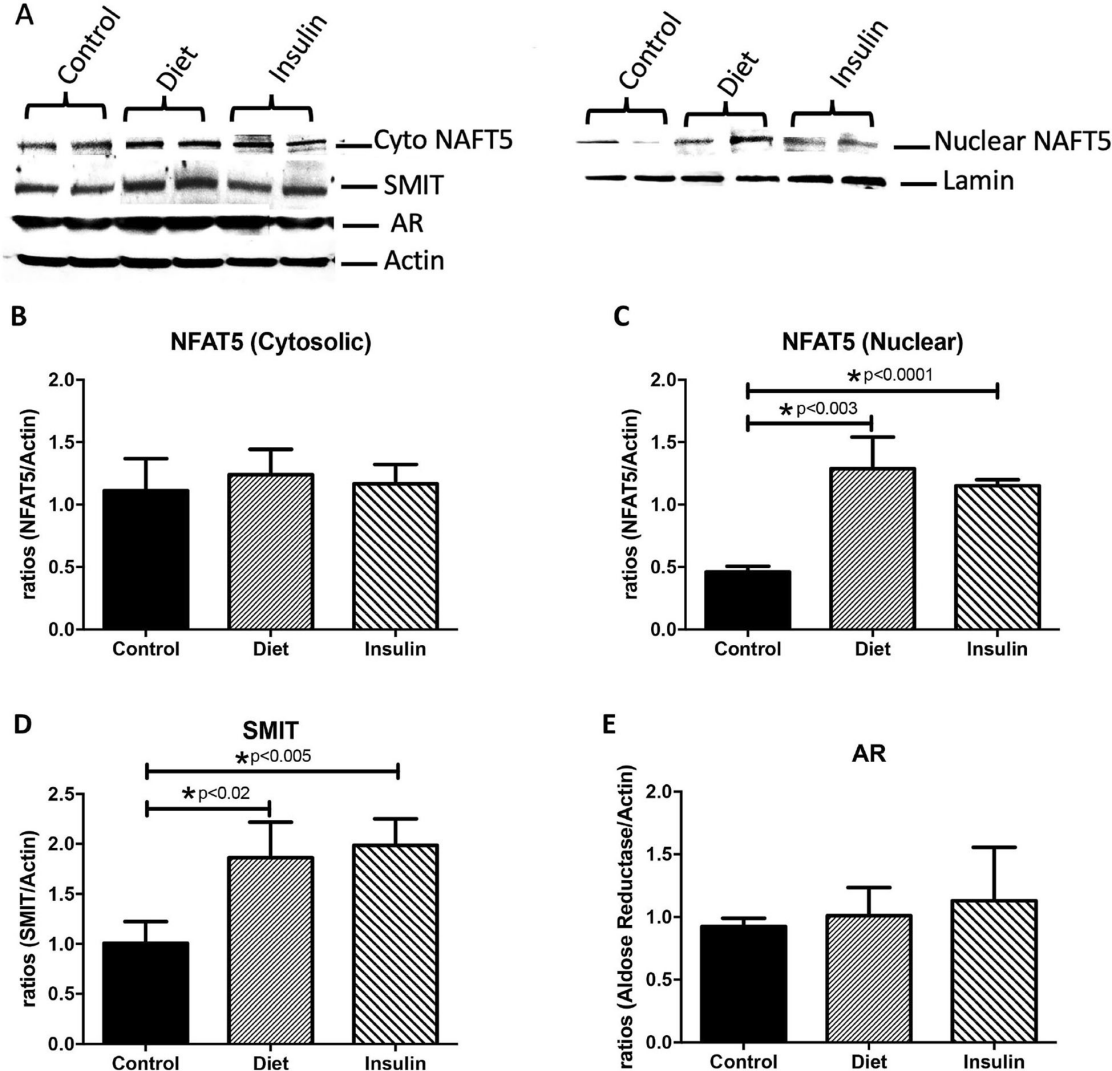
NFAT5, SMIT and AR in control and GDM human placentas. Cytosolic and nuclear levels of NFAT5, SMIT and AR (*n* = 5) were measured by western blot and quantified by Spot Denso analysis. Characteristic western blots for NFAT5, SMIT and AR are shown in (**a**). Cytosolic NAFT5 levels were not changed in the GDM-D or the GDM-I placentas when compared to control samples (**b**). Nuclear NAFT5 levels were elevated in in both GDM-D and GDM-I (*p* < 0.05) placenta when compared to control placenta samples (**c**). Cytosolic SMIT was increased in both GDM-D and GDM-I placenta as compared to controls (**d**). There were no changes for AR expression between control and GDM placentas (**e**). Experiments were conducted in triplicate and statistically different values are noted as * *p* < 0.05

Studies have shown that an increase in osmolarity leads to the activation of TonEBP/NFAT5 [22]. Activation of TonEBP/NFAT5 leads to increased expression of transmembrane proteins such as sodium-dependent myo-inositol transporter (SMIT) as well as the induction of the aldose reductase enzyme (AR; responsible for sorbitol production), which regulates the production and accumulation of inositol and sorbitol. Collectively, these factors regulate production and transport of organic osmolytes into cells to maintain normal osmolarity and cell volume [22]. Figure 3a shows a characteristic western blot for NFAT5, SMIT and AR of treated trophoblast cells as compared to controls. We first investigated the cytosolic and nuclear expression of NFAT5 in the human placenta of control and GDM patients. We observed increased expression of nuclear NFAT5 in both GDM-D (2.8-fold; *p* < 0.003) and GDM-I (2.5-fold; *p* < 0.0001), but cytosolic NAFT5 was not elevated in the GDM placentas when compared to controls (Fig. 3b, c). A significant increase in SMIT was observed in the GDM-D (1.8-fold; *p* < 0.02) and GDM-I (2-fold; *p* < 0.005) placenta when compared to controls (Fig. 3d). No expression differences were observed for AR when comparing GDM and control placentas (Fig. 3e).

### Active caspase 3 and XIAP

**Fig. 4:**
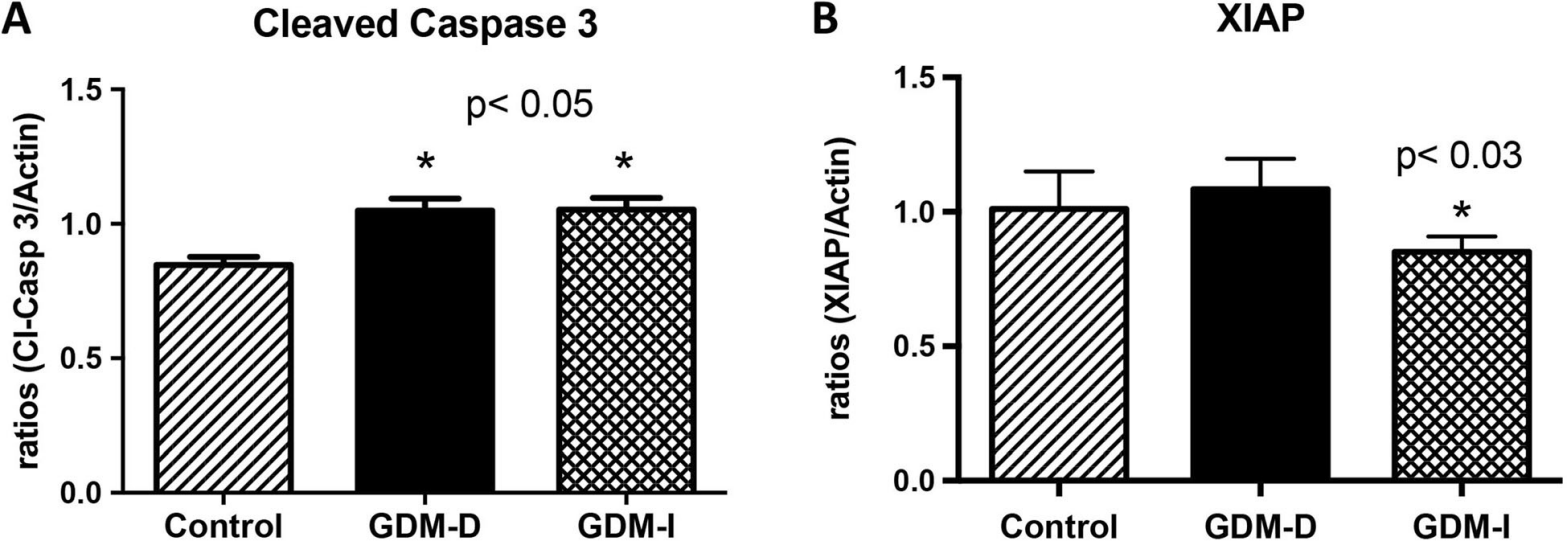
Cleaved caspase 3 and XIAP expression in control and GDM human placentas. Levels of cleved caspase 3 and XIAP (*n* = 5) were measured by western blot and quantified by Spot Denso analysis; histograms show mean ± SEM. Cleaved caspase 3 was elevated in both the GDM-D and GDM-I placentas (*p* < 0.05) when compared to controls (**a**). XIAP protein was decreased only in the GDM-I placentas (*p* < 0.03) when compared to controls (**b**). Experiments were conducted in triplicate and statistically different values are noted as * *p* < 0.05

Decreased apoptosis is present in the GDM placenta when compared to control placentas [3]. Active caspase 3 and the anti-apoptotic inhibitor of caspase XIAP was evaluated in the placenta of control and diet or insulin treated GDM patients. Specifically, there was an upregulation of active caspase 3 (1.2-fold; *p* < 0.05) in the placentas from both GDM-I and GDM-D when compared to control placental tissue (Fig. 4a). Interestingly, a significant decrease of XIAP expression (1.7-fold; *p* < 0.05) was only observed in the GDM-I placenta when compared to controls (Fig. 4b).

### Insulin and ceramide inhibition of villi trophoblast mitochondrial respiration

**Fig. 5:**
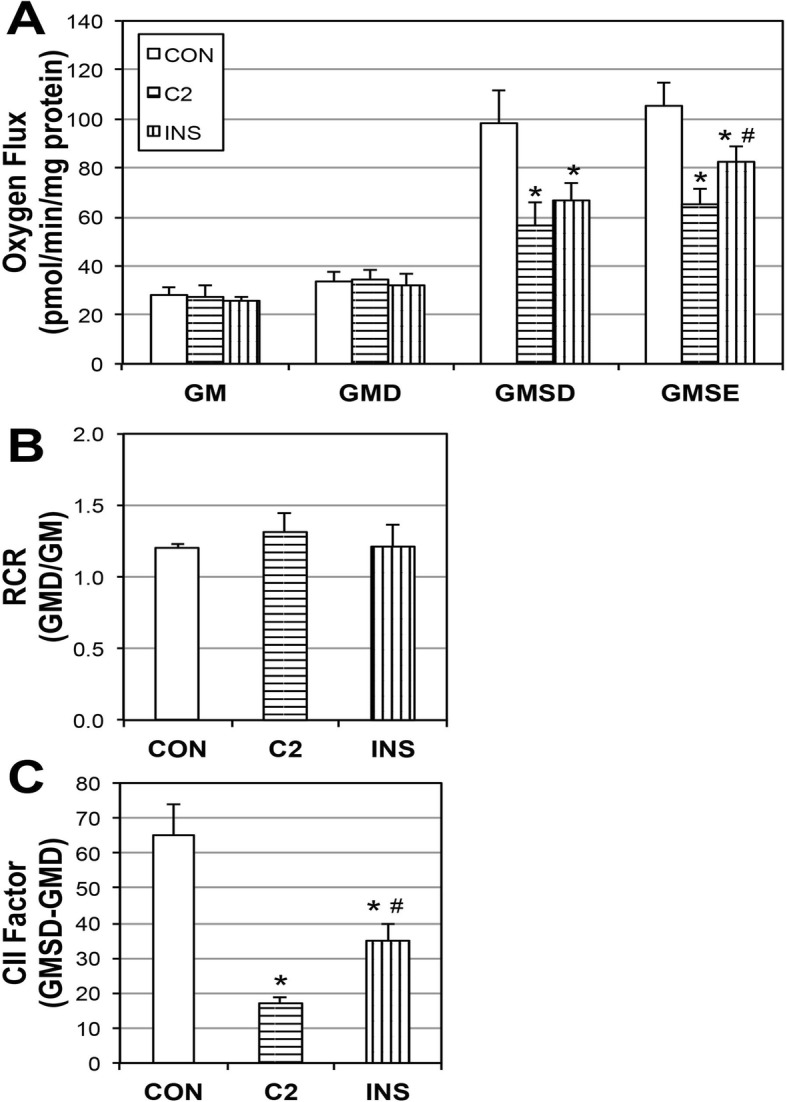
Ceramide and insulin reduce mitochondrial respiration. BeWo choriocarcinoma cells were treated with insulin (INS; 50 nM) or C2-ceramide (C2; 1 μM) for 16 h (*n* = 3). To measure mitochondrial respiration (**a**), cells were treated with: GM, Glutamate (10 mM) + Malate (2 mM); GMD: + ADP (2.5 mM); GMSD, + Succinate (10 mM); GMSE, + FCCP (0.05 μM). Respiratory control ratio (RCR; (**b**)) and Complex II Factor (CII Factor; (**c**)) were determined by the analysis indicated. *p < 0.05 for condition vs. controls (CON). #*p* < 0.05 for condition vs. C2

**Fig. 6:**
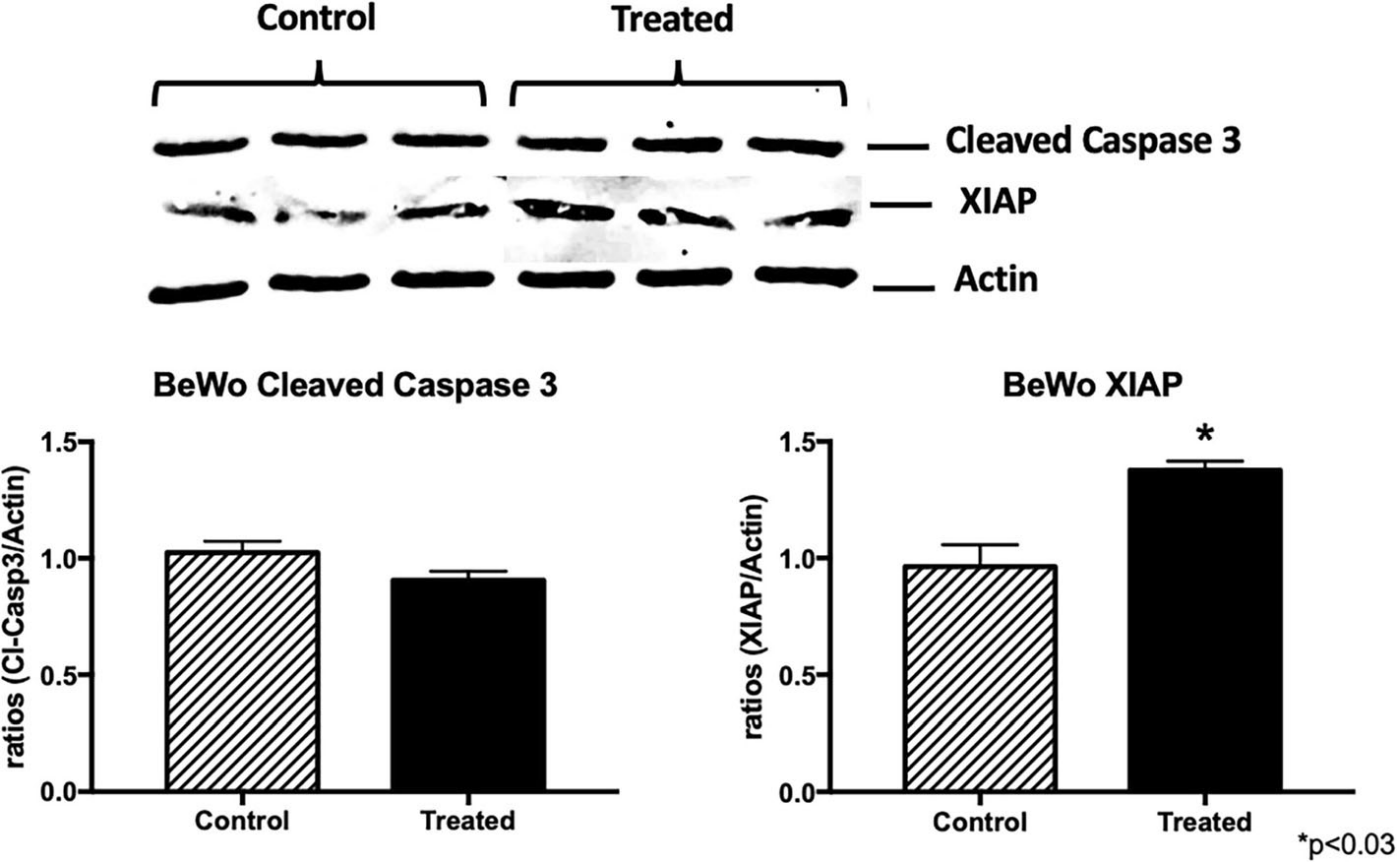
Cleaved caspase 3 and XIAP expression in control ceramide treated trophoblast cells. Levels of cleved caspase 3 and XIAP (*n* = 4) were measured by western blot and quantified by Spot Denso analysis; histograms show mean ± SEM. Cleaved caspase 3 was not changed in treated cells when compared to controls (**a**). XIAP protein was increased in the treated cells (*p* < 0.03) when compared to controls (**b**). Experiments were conducted in triplicate and statistically different values are noted as * *p* < 0.05

To provide further evidence of altered cellular function, and to mimic the pregnancy milieu of GDM, we treated human placental trophoblast villi cells (BeWo) with insulin (50 nM) or ceramide (C2-ceramide; 1 μM), as used previously [23], prior to placement into respirometer chambers. Oxygen flux was determined in conditions of multiple substrates (Fig. 5a; see methods or legend for details). Both treatments resulted in a significant reduction in mitochondrial respiration compared with controls, which became apparent upon the addition of succinate (GMSD) and remained with addition of FCCP (GMSE). Despite the difference in respiration rates across treatments, respiratory control ratios (RCR; Fig. 5b), a general indicator of mitochondrial function, revealed no apparent differences in the functionality or overall health of the mitochondria. Lastly, the profound disparity across treatments in response to succinate (GMS) was very apparent when we determined the complex II factor, an indicator of succinate sensitivity (Fig. 5c), wherein C2 and insulin (INS) treatments were significantly lower vs. controls (CON), albeit to varying degrees. Active caspase 3 and the anti-apoptotic inhibitor of caspase XIAP was also evaluated in control and ceramide treated BeWo cells. There was no significant change in active caspase in the ceremide treated trophoblast when compared to control placental tissue (Fig. 6). Interestingly, a significant increase of XIAP expression (1.7-fold; *p* < 0.03) was observed in the treated trophoblasts when compared to controls (Fig. 6).

## Discussion

Although GDM is developed during pregnancy, its effects promulgate even after birth. Women who experience GDM during their pregnancies are at increased risk of developing type 2 diabetes mellitus (T2DM) in the years following delivery. Children of GDM-affected mothers are likewise at a higher risk of developing T2DM and of being obese [24]. The etiology of GDM has not been fully elucidated, but the pathophysiology of the disease arises from insulin resistance. Diagnoses of GDM usually come after a screening of high-risk patients at 24–48 weeks of gestation. Patients at risk of GDM usually have a family history of GDM (or any other type of diabetes) and/or a previous pregnancy with a macrosomic infant [25]. Although early intervention could play a positive role in outcome, current research in the field of placental abnormalities is attempting to identify plausible pathophysiological mechanisms that contribute to this disease in order to learn to prevent and even intervene in its progression.

Successful pregnancies require maternal tissues to become progressively insulin resistant. Although the cause of this insensitivity is unknown, it is thought to be orchestrated by placental hormones and other factors that are expressed during pregnancy yet are not fully characterized [24]. Since GDM is observed in both obese as well as lean women, different pathophysiological pathways have been proposed in the development of the disease. Although lean women experience similar physiological mechanisms, it is thought that GDM has a larger role in the first-phase insulin response. Since the elevated levels of maternal glucose during GDM are transferred to the fetus through the placental intervillous space, maternal hyperglycemia stimulates fetal hyperinsulinemia. High levels of insulin circulating through the body of a fetus causes a stimulation of growth known as macrosomia [24].

Our focus on ceramides as a potential mediator of GDM-placental complications stems from our previous work that reveals ceramide accumulation being a tangible outcome of inflammation [26, 27] and hyperinsulinemia [17, 28]. That the placenta from insulin-treated patients was more greatly enriched with ceramides supports our previous work of the biosynthetic effects of insulin on ceramides. More interestingly, we observed high ceramide levels in the nucleus of the syncytiotrophoblast from GDM patients treated with insulin. This expression correlated with higher expression of nuclear SPT, the rate-limiting enzyme of ceramide biosynthesis. Although such nuclear levels of ceramide were unexpected, this localization had been observed in other systems where it is correlated with apoptosis signaling [29]. Hyperosmolar stress is one potential reason for the accumulation of polyols in placental and fetal tissues [30]. Recent studies showed a correlation between hyperosmolarity and increased Ceramide production [12]. Increases in osmolarity leads to activation of NFAT5 by phosphorylation and, subsequently, to nuclear translocation [22]. Tellingly, we observed increased NFAT5 in the nucleus of the placental cells of the GDM patients. This suggests the presence of abnormal osmolarity during this condition and supports the idea that hyperosmolarity may have a role in the increased nuclear ceramide level observed during GDM. Together with increased NFAT5, upregulated SMIT suggests a role of inositol in response to the hyperosmolar environment during GDM that requires further careful investigation.

In our system, we observed increased activation of cleaved caspase 3 in the placenta of insulin-treated GDM. These results were correlated with decreased XIAP, a known inhibitor of caspase 3 activation. These two discoveries highlight a novel intersection between nuclear placental ceramide level and the control of apoptosis signaling in GDM treated with insulin. Our previous work has implicated both insulin and ceramides in the disrupted mitochondrial states coincident with metabolic pathologies, particularly in skeletal muscle and heart [17, 28]. Furthermore, BeWo studies showed increased mitochondrial bioactivity in hypoglycemic environments [31]. Our current findings are extensions of that work as data reveal that both insulin and ceramides specifically elicit potentially adverse mitochondrial anomalies in cells of placental relevance. Interestingly, we did not observe activate caspase 3 in the cells treated with ceramide. This was unexpected considering previous work that has revealed ceramide as an inducer of apoptosis. This could be explained by the fact that we observed an increase in XIAP protein a known inhibitor of caspase 3 activation.

Multiple studies have found correlations between GDM and differential trophoblast behavior. Indeed, hyperglycemia affects the behavior of trophoblast cells, as shown by Cawyer et al. [32]. Other data show that as hyperglycemia increases, various cytokines (including IL-6) are augmented and a number of growth factors including vascular endothelial growth factor (VEGF) and placental growth factor (PlGF) are inhibited [32]. Ultimately these angiogenic and vasculogenic growth factors are necessary for efficient remodeling and vascularization of the placenta. Accordingly, their demise during hyperglycemic conditions demonstrates a negative effect of glucose on trophoblast biology.

The conventional treatments for GDM are alterations in diet, physical activity, and insulin therapy. While insulin therapy is effective at controlling glucose, increasing evidence suggests that many of the consequences of insulin resistance states, such as GDM and T2DM, begin prior to meaningful changes in glycemia, suggesting a greater relevance for hyperinsulinemia and insulin resistance over hyperglycemia. These consequences include increased cardiovascular disease mortality [33], increased cancer mortality [34], and exacerbated insulin resistance [34]. The exaggerated insulin may also promote excessive maternal and fetal fat gain [34, 35]. Though our findings strongly implicate a relevance for ceramides, future studies will elucidate the roles of specific ceramide species (i.e., chain length). Nevertheless, our results of increased placental ceramide accrual and altered mitochondrial function add new data that suggest additional caution related to the use of insulin therapy in GDM.

## Conclusions

Our findings confirm the presence of ceramide in the human placenta of control and GDM patients. Furthermore, our results demonstrate that ceramide is increased in the placental trophoblast during insulin treatment and that this upregulation correlates with increased hyperosmolarity, increased caspase activation and decreased XIAP. Our results further suggest that increased activation of placental caspase 3 during GDM-D is occurring in a ceramide independent manner. Treatment variation between diet and insulin suggests that an equally effective but alternative mechanism is exerted in the placenta leading to the alleviating of the symptoms and/or consequences present in the placenta during GDM.

## Data Availability

Data and other materials are available from the corresponding author on reasonable request.

## Abbreviations

AR: Aldose reductase
GDM: Gestational diabetes mellitus
NFAT5: Nuclear factor of activated T cells
SMIT: Sodium myo-inositol transporter
SPT1: Serine palmitoyltransferase 1
XIAP: X-linked inhibitor of apoptosis
